# Editing DNA Methylation in Mammalian Embryos

**DOI:** 10.3390/ijms21020637

**Published:** 2020-01-18

**Authors:** Taiga Yamazaki, Yu Hatano, Ryoya Taniguchi, Noritada Kobayashi, Kazuo Yamagata

**Affiliations:** 1 Division of Biomedical Research, Kitasato University Medical Center, Kitasato University, 6-100 Arai, Kitamoto, Saitama 364-8501, Japan; kenchu@insti.kitasato-u.ac.jp; 2 Faculty of Biology-Oriented Science and Technology, KINDAI University, 930 Nishimitani, Kinokawa, Wakayama 649-6493, Japan; 1944710002d@waka.kindai.ac.jp (Y.H.); 1933710007k@waka.kindai.ac.jp (R.T.)

**Keywords:** DNA methylation, epigenome editing, preimplantation embryo, germ cell, centromere, pericentromere

## Abstract

DNA methylation in mammals is essential for numerous biological functions, such as ensuring chromosomal stability, genomic imprinting, and X-chromosome inactivation through transcriptional regulation. Gene knockout of DNA methyltransferases and demethylation enzymes has made significant contributions to analyzing the functions of DNA methylation in development. By applying epigenome editing, it is now possible to manipulate DNA methylation in specific genomic regions and to understand the functions of these modifications. In this review, we first describe recent DNA methylation editing technology. We then focused on changes in DNA methylation status during mammalian gametogenesis and preimplantation development, and have discussed the implications of applying this technology to early embryos.

## 1. Introduction

Cytosine methylation is a process in which methyl groups are added to the cytosine of CpG dinucleotides, forming 5-methylcytosine (5mC). This epigenetic modification of DNA plays crucial roles in various developmental process such as X-chromosome inactivation, and in genome imprinting through regulation of transcription [1]. During development, there are dynamic changes in DNA methylation in that established DNA methylation patterns in cells—specific to spermatozoa and oocytes—are reprogrammed after fertilization, and tissue- or cell-type-specific DNA methylation patterns are formed. Moreover, the accumulation of abnormal DNA methylation leads to diseases and developmental disorders [2].

Two types of enzymes are involved in the establishment or maintenance of DNA methylation. One group includes the de-novo-type DNA methyltransferases DNMT3A and DNMT3B, which are responsible for establishing DNA methylation. Another type of DNA methyltransferase is DNMT1—a maintenance type of enzyme. It works at hemimethylated CpG sites after DNA replication. Conversely, there have also been reports of DNA-demethylation-related enzyme, called ten-eleven translocation (TET) methylcytosine dioxygenase, which works in oxidation of 5-methylcytosine and creating 5-hydroxylmethylcytosine.

To understand the function and importance of DNMTs or TET proteins, gene knockout (KO) mice were generated and analyzed. Because *Dnmt1*- or *Dnmt3a/3b*-deficient mice die during embryonic development, the establishment and maintenance of DNA methylation are clearly essential [3,4]. It has also been reported that *Tet1*-deficient mice reveal inefficient erasure of genome imprinting in primordial germ cells (PGCs) and defects in mitotic gene expression [5,6]. In addition, conditional KO mice of *Dnmt3a/3b* were also created. These studies provided great insights into the regulation of genomic imprinting during germ cell formation, and the reprogramming of DNA methylation after fertilization [3,4,5,7,8]. From another point of view, *Dnmt/Tet* gene KO is not only a technique for analyzing the functions of DNMT/TET molecules, but is also a method used for regulating genome-wide DNA methylation. However, techniques for manipulating DNA methylation targeting only specific genomic loci are not well established, and this limitation has become a bottleneck in analyzing the functions of nongenomic information. Epigenome editing is a technology that can help to eliminate this bottleneck. Because, in many cases, the transcriptional activity of target genes can be controlled by such editing, medical applications are anticipated for treating dysfunctions caused by abnormal gene expression [9]. It is also possible to analyze not only transcriptional regulation by DNA methylation, but also new noncoding DNA (ncDNA) functions arising via DNA methylation, such as the physical structure or signaling platforms of genomes [10].

In this review, methods for editing DNA methylation in mammalian cells are outlined first. We then summarize recent efforts towards editing DNA methylation patterns in early embryos and discuss the significance of these techniques.

## 2. Editing DNA Methylation

Genome editing using molecules such as zinc finger nuclease (ZFN) [11], transcription activator-like effector nuclease (TALEN) [12], and clustered regularly interspaced short palindromic repeats (CRISPR)/CRISPR-associated protein 9 (Cas9) [13], are technologies that induce gene disruption by generating double-stranded DNA breaks in target sequences. In ZFN and TALEN, DNA binding modules such as zinc finger (ZF) or transcription activator-like effector (TALE) are fused with Fok I DNA nuclease, which is activated by dimerization of another pair of Fok I fused with ZF or TALE. These artificial nucleases lead double-stranded DNA breaks. In the CRISPR system, Cas9 is nuclease and it recognizes DNA sequences with target-specific guide RNA (gRNA). Because it is possible to bring molecules to bind to arbitrary DNA sequences, the application of such genomic editing to other techniques such as genomic imaging using fluorescent proteins [14], transcriptional control using transcription activators or repressors [15], and the identification of chromatin-binding molecules [16] has been reported. To edit the epigenome, epigenetic modifying enzymes serving as effector proteins are fused with these DNA-binding modules. Various studies on editing DNA methylation have been published [17]. In most cases, DNMT or TET1 are fused with DNA-binding modules such as zinc finger (ZF), transcription activator-like effector (TALE), or catalytically dead Cas9 nuclease (dCas9) for editing DNA methylation. 

### 2.1. Induction of DNA Methylation

**Figure 1:**
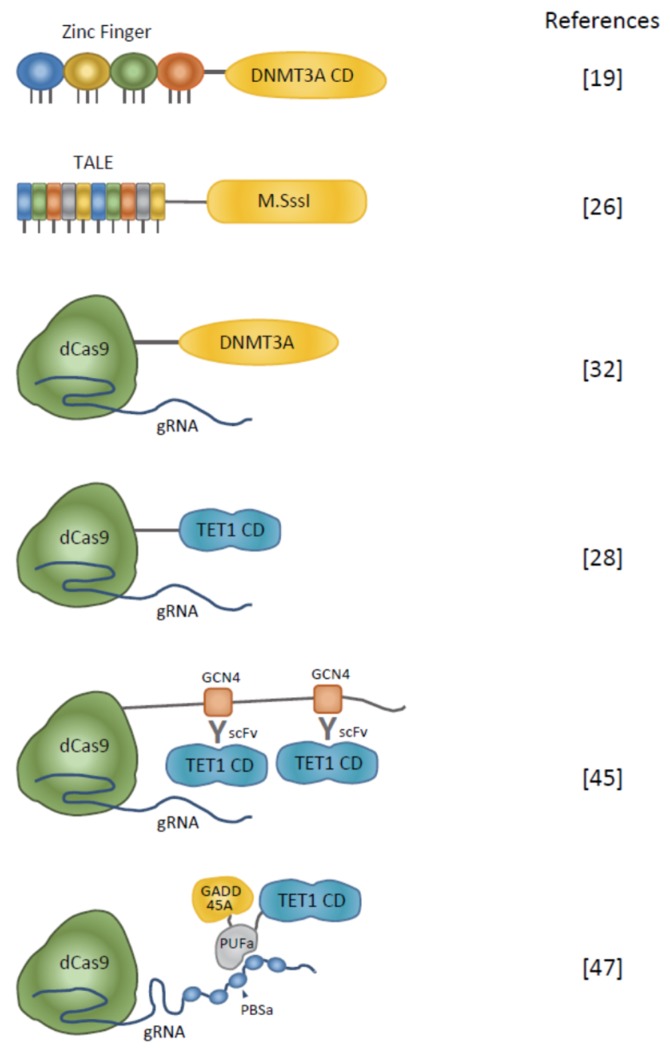
Schematic diagrams of artificial enzymes for editing DNA methylation. Representative combinations of DNA-binding modules and effectors are shown. Zinc finger, transcription activator-like effector (TALE), and dCas9 with guide RNA (gRNA) complex are used for DNA-binding modules. DNMT3A or M.SssI are effectors of inducing DNA methylation. To remove DNA methylation, the catalytic domain (CD) of TET1 is fused with DNA binding module. SunTag technology enables multiple copies of TET1 CD to be introduced to the target region [41]. There has been a report describing the tethering of both TET1 CD and base excision repair (BER)-related proteins such as GADD45A to improve the efficiency of DNA demethylation [42].

Human DNMT3A has been most frequently used as an effector to introduce DNA methylation [17] (Figure 1 and Table 1), and there have been few examples of using DNMT1 for editing DNA methylation. DNMT3A is a de novo type of DNA methyltransferase that induces DNA methylation in a DNA-replication-independent manner and helps to establish it. This function is distinguished from the maintenance type of DNA methylation, which is coupled to DNA replication and ensured by DNMT1. DNMT3A consists of a regulatory region containing the *N*-terminal PWWD domain, ADD domain, and a *C*-terminal catalytic domain. Because it is a large molecule (912 amino acids, aa), only the catalytic domain is used frequently [17], and ZF [18,19,20,21,22,23], TALE [24,25,26], and dCas9 [25,27,28,29,30,31,32] have been fused with the catalytic domain of DNMT3A. There have been reports on the optogenetic regulation of enzymatic activity of epigenome-editing enzymes. Thus, optogenetic-related proteins CIB1 and CRY2 (a blue light-inducible dimerizing protein pair), were fused with TALE and effector proteins, respectively. TALE–CIB1 and TET1–CRY2 (DNMT3A–CRY2) dimerize by blue light and allow editing of DNA methylation on the *Ascl1* gene [26] (Table 1). DNMT3A interacts with its enzymatically inactive cofactor DNMT3L, and this interaction stimulates the enzymatic activity of DNMT3A [33,34,35]. The DNMT3A and DNMT3L fusion protein has been used as an effector domain of epigenome-editing enzymes [19,24]. To increase the interaction of DNMT3A and target sequences, there has been an attempt to recruit multiple copies of DNMT3A to target sequences. In this regard, the SunTag system was applied for editing DNA methylation and multiple GCN4 peptides were fused with the dCas9 protein. Because GCN4 is recognized by a single-chain variable fragment (scFv), multiple DNMT3A molecules fused with scFv were recruited by a region tethered by dCas9–GCN4 peptides [31] (Table 1). In addition, another example used MIWI2, a mouse P-element-induced wimpy testis (PIWI)-related protein, as an effector of methyltransferase activity. Thus, MIWI2 has crucial roles for the de novo methylation of retrotransposons via interactions with PIWI-interacting RNAs (piRNAs) in spermatogenesis [36]. ZF–MIWI2 targeted to Line1 retrotransposon induces DNA methylation in male germ cells [23] (Table 1).

While mammalian DNA methyltransferase is frequently used as the effector protein for epigenome-editing enzymes, the bacteria-derived methyltransferase M.SssI has also been used as an effector protein for introducing DNA methylation [25,29,30] (Table 1 and Figure 1). Compared with DNMT3A, SssI is a small molecule (386 aa) present in *Spiroplasma* spp. [37]. SssI does not require a binding partner for its enzymatic activity, unlike DNMT3A, and the purified protein is available commercially. Additionally, the functions of the amino acid residues of SssI have been well studied [38,39]. Because SssI is a strong DNA methyltransferase, it helps to regulate enzymatic activity by controlling the expression of genes [25], using enzymatically optimized SssI mutants [29] or enzyme splitting [30] to decrease off-target effects. There are some advantages to the use of SssI as an effector protein of epigenome-modifying enzymes. When using DNMT3A as an effector, it is possible that DNA methylation is inefficient when applied to cells expressing DNMT3L at very low levels, such as testis-derived germline stem cells (GS cells) [40]. In such cases, applying the SssI system seems to be effective. In addition, it is also possible that the application of SssI is effective for introducing DNA methylation into species that do not have the DNMT system.

### 2.2. Erasure of DNA Methylation

TET1 is a chromatin-modifying enzyme mainly used to induce DNA demethylation. Upon oxidation by TET1, 5mC is converted to 5-hydroxymethylcytosine (5hmC), 5-formylcytosine (5fC), and 5-carboxylcytosine (5caC), and the base excision repair system finally converts 5fC or 5caC to C [43]. When TET1 is used as an effector protein for DNA demethylation, as in DNMT, its catalytic domain is frequently used in epigenome editing [9,26,28,32,44,45,46] (Figure 1 and Table 2). It has also been reported that the catalytic domain of TET2 is fused with Zinc Finger [41]. The application of the SunTag system to tether multiple TET1 proteins to increase the efficiency of DNA demethylation in the target sequence has also been reported [47] (Figure 1 and Table 2). Morita et al. reported no significant difference in DNA demethylation activity between dCas9–TET1 and dCas9–catalytically dead Tet1 (dTet1) on the *Gfap* and *H19* loci, whereas the dCas9–SunTag system, which enables the tethering of multiple copies of TET1, has strong DNA demethylation activity [47]. On the other hand, other groups have reported that the dCas9–TET1 system is sufficient to decrease DNA methylation in target loci [9,28,32,45,46]. It is possible that the number of TET1 molecules sufficient for demethylation activity varies depending on the locus. Chen et al. developed a versatile CRISPR/Cas9 platform called “Casilio”, consisting of a CRISPR–Cas9–Pumilio hybrid. In this system, the effector protein is fused with the Pumilio/FBF RNA-binding domain (PUF domain) and a DNA-binding module comprising a dCas9 and gRNA complex in which multiple PUF binding sites are expressed together with single guide (sg) RNA [42]. Because the PUF domain binds specific 8 mer RNA sequences of PUF binding sites, this system enables multiple PUF–effector fusion proteins to bind to target loci. Taghbalout et al. reported increased efficiency of DNA demethylation and derepression of genes by multiple tethering of TET1 by a Casilio complex named “Casilio–ME1” compared with the efficiency of DNA demethylation by the dCas9–SunTag system [48]. They also reported a trial of tethering base excision repair (BER)-related proteins GAAD45A or NEIL2 together with TET1, expecting increased removal of oxidized cytosine intermediates (Figure 1 and Table 2). These systems, named “Casilio–ME2” (using GAAD45A) and “Casilio–ME3” (using NEIL2), showed great increases in DNA demethylation and gene activation in target loci compared with Casilio–ME1, which simply tethers multiple TET1 molecules to the target sequence.

## 3. Evaluation of DNA Methylation

To study DNA methylation, it is necessary to detect its status in specific cells. Immunostaining using anti-5mC antibodies, methylation-specific polymerase chain reaction (MSP), and bisulfite sequencing have been used widely to analyze DNA methylation [49]. Recently, with the rise of next-generation sequencers, methylated DNA immunoprecipitation-sequencing (MeDIP-seq) and whole-genome bisulfite sequencing (WGBS), which enable high-throughput analysis of DNA methylation, have been developed [50]. In addition, it is possible to detect genome-wide DNA methylation in living cells with fluorescent probes specific for DNA methylation [51,52]. In these studies, the methyl CpG binding domain (MBD) of human methyl-CpG-binding protein 1 (MBD1) containing a nuclear localization signal (NLS) was fused with a fluorescent protein (FP) such as EGFP or mCherry in the *N*-terminus of the MBD. This FP–MBD–NLS complex binds methylated DNA specifically and can detect the genome-wide DNA methylation status in various types of living cells. This technique enables an easy comparison of the DNA methylation status of cells, and has shown that somatic cells and tissues have high levels of DNA methylation, whereas germ cells show hypomethylation in centromeres and pericentromeres [53].

## 4. Editing DNA Methylation in Mammalian Embryos

Much of the research on DNA methylation editing has been conducted in cultured cells. The application of DNA methylation editing in vivo has also been reported for human clinical therapy in fetal and adult brains [9,47]. On the other hand, there have been few reports about the application of DNA methylation editing to embryos or germ cells. Wei et al. have successfully manipulated the methylation status of intracisternal A-particle (IAP), a repetitive sequence derived from a retroviral element, acting as a promoter located upstream of the *Agouti* gene. To do this, they used dCas9–DNMT3A or dCas9–TET1 in germinal vesicle (GV)-stage oocytes (premeiotic-stage oocytes before ovulation) [32]. This methylation editing resulted in changes in the coat color of offspring. Because the IAP promoter affects expression of the *Agouti* gene, and its activity is dependent on DNA methylation [54], targeting DNA methylation to the IAP promoter derived from a mother with a yellow coat resulted in the birth of offspring with pseudoagouti coat color. By contrast, removal of DNA methylation from the IAP promoter of GV-stage oocytes derived from pseudoagouti mothers resulted in offspring with yellow coats [32]. In 2004, Kono et al. succeeded in producing viable bimaternal mice by nuclear transfer of nongrowing (ng) and fully grown (fg) oocytes with enhanced *Igf2* expression, by targeted disruption of the *H19* DMR [55]. This is a modification of genome imprint by genetic engineering. Wei et al. repeated this experiment and were able to produce bimaternal mice by epigenome editing. Induction of DNA methylation in the *H19* ICR and *Dlk1-Dio3 IG*-DMR regions among embryos reconstructed from ng and fg oocytes by nuclear transfer resulted in viable offspring [32].

Application of the bacterial CpG methyltransferase SssI to oocytes has also been reported. Lei et al. introduced DNA methylation at the CTCF, a transcriptional repressor, binding upstream of *H19* by injecting plasmids encoding dCas9–SssI (Q147L, a point mutant in which the glutamine-147 of SssI was replaced with leucine) together with gRNA into pronuclear-stage oocytes [29]. They confirmed the introduction of DNA methylation by analyzing the genome of tail tissues of the offspring produced by transplantation of oocytes injected with dCas9–SssI, suggesting that the artificial methylation in this site was maintained from oocytes to adult mice.

## 5. Features of DNA Methylation in Mouse Germline Cells

**Figure 2:**
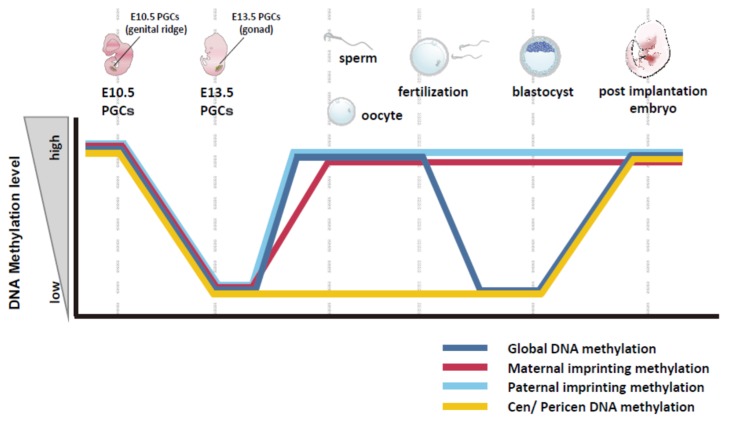
Dynamics of DNA methylation in mouse germline cells. Global DNA methylation (blue), maternal imprinting methylation (red), paternal imprinting methylation (light blue), and centromeric and pericentromeric DNA methylation (yellow) were erased in primordial germ cells (PGCs) at embryo days embryonic day (E) 10.5–E13.5 by both active and passive DNA demethylation. These regions acquire DNA methylation during spermatogenesis and oogenesis and reveal genome-wide hypermethylation, whereas centromeric and pericentromeric DNA sequences undergo hypomethylation. After fertilization, genome-wide DNA demethylation occurs during preimplantation development. Both paternal and maternal imprinting regions are protected from this DNA demethylation, and centromeric and pericentromeric DNA methylation patterns retain their hypomethylated status. After implantation and during cellular differentiation, global DNA and centromeric and pericentromeric DNA sequences become hypermethylated.

There are two unique steps of global DNA demethylation and remethylation in germline cells during development. One is DNA demethylation—so-called genomic reprogramming—in preimplantation development and subsequent methylation of specific gene loci after implantation; the other is establishment of germ-cell-specific DNA methylation patterns during germ cell formation and gametogenesis, resulting in genomic imprinting [56,57] (Figure 2).

DNA methylation profiles differ among adult tissues and cell types, in what are called tissue-specific differentially methylated regions (tDMRs) [58], and it has been reported that differentiated tissues and cells generally show genome-wide high DNA methylation [59]. Spermatozoa and oocytes are also hypermethylated, but genome-wide DNA methylation is largely erased through preimplantation development [60]. This is called reprogramming of DNA methylation, and 5mC is demethylated via 5hmC in the male pronucleus after fertilization by oxidation of 5mC by the Tet3 enzyme [8]. In addition, lack of maintenance of DNA methylation with Dnmt1 also contributes to DNA demethylation during preimplantation development in conjunction with demethylation by Tet3 [61]. After implantation, the genome-wide DNA methylation pattern changes from hypomethylation to hypermethylation [62]. Focusing on imprinted genes, the DNA methylation of key imprinting control regions escapes from DNA demethylation during preimplantation development and maintains its imprinted patterns [63,64,65].

Germ cells originate from PGCs, which are induced by stimulation of cytokines such as bone morphogenetic protein (BMP)-4 secreted from mouse embryonic ectoderm at embryo day (E) 6.0 [66]. PGCs show genome-wide hypermethylation and maintain genomic imprinting. During mouse development from E10.5 to E13.5, genome-wide DNA demethylation occurs and erases the genomic imprint of DNA methylation in PGCs [67,68]. This DNA demethylation is caused by both Tet1 [6] and replication-coupled passive DNA demethylation [69]. After that, imprinting patterns of DNA methylation are established by *Dnmt3a*, *Dnmt3b*, and *Dnmt3l* during gametogenesis [7,70].

Apart from DNA methylation of imprinted genes, ncDNA sequences also show unique aspects of methylation during germ cell formation and preimplantation development. Centromeres comprise ncDNA and are essential for chromosome segregation in mitosis [71]. In the mouse, centromeres consist of tandem repeat sequences called minor satellites, and they serve to form kinetochore structures with binding of centromere/kinetochore proteins via microtubules. Adjacent to the centromere is the pericentromere region consisting of another repetitive sequence called the major satellite. These two repeats—the minor and major satellites—have unique epigenetic features. Major satellites have repressive epigenetic marks such as DNA methylation and trimethylation of histone H3 at lysine 9 (H3K9me3), and minor satellites have a centromere-specific variant of histone H3, CENPA [71]. It has been reported that centromeres and pericentromeres in the spermatozoa and oocytes are hypomethylated in the mouse, even though they have genome-wide hypermethylation status in somatic cells [53]. After fertilization, both centromeres and pericentromeres maintain their hypomethylation status during preimplantation development, and they then show a transition from hypomethylation to hypermethylation after implantation. During the phase of DNA demethylation in mouse PGCs from E10.5 to E13.5, DNA demethylation occurs both in centromeres and pericentromeres, and this hypomethylation is maintained during spermatogenesis. Interestingly, this hypomethylation of centromeres and pericentromeres in mouse germline cells is also commonly observed in human spermatozoa [72,73]. The biological significance of the germ-cell-specific features of DNA methylation in functional regions of the chromosomes and an enormous region accounting for a few percent of the genome—centromeres and pericentromeres—is still unclear. To answer this question, it will be necessary to manipulate DNA methylation in a target-specific manner in living cells and mice and analyze the outcomes. In this regard, epigenome editing offers a feasible approach for manipulating the DNA methylation of target regions in germline cells.

## 6. Artificial Introduction of DNA Methylation into the Pericentromeres of Mouse Embryos

**Figure 3:**
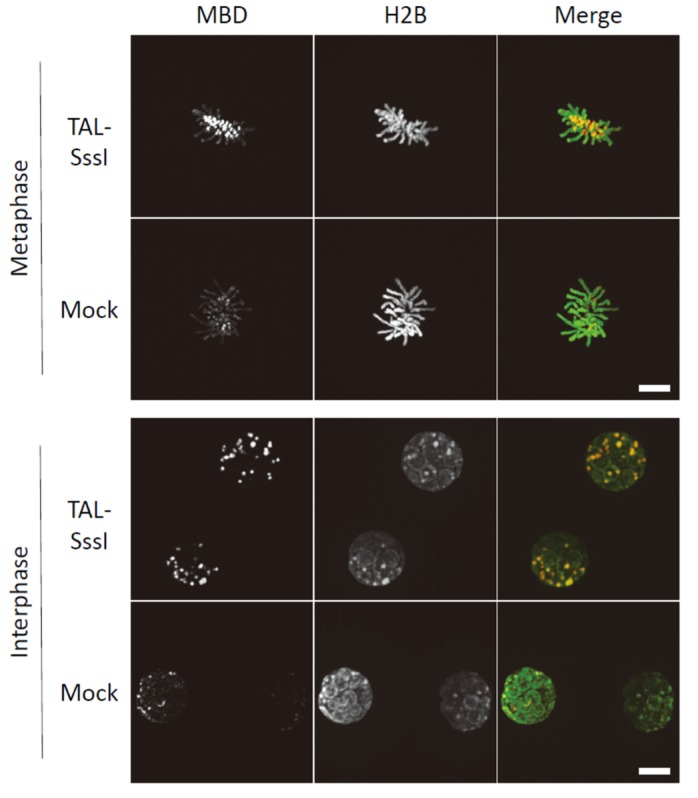
Induction of DNA methylation in the pericentromeres of mouse embryos. Pericentromeric (major satellite) DNA methylation is upregulated by targeted DNA methyltransferase, TALMaj–SssI (TAL–SssI) in mouse embryos. Fertilized embryos were labeled with a DNA methylation marker (mCherry–MBD–NLS: MBD, red) and a chromatin marker (histone H2B–EGFP: H2B, green). “Mock” represents embryos injected only with mCherry–MBD–NLS and histone H2B–EGFP. “Metaphase” chromosomes are shown as snapshots of chromosomes at syngamy, and “Interphase” images are of two cell embryos. Note that the images of TAL–SssI-expressing embryos represent the highly DNA methylated status in pericentromeres and heterochromatin compared with controls. Scale bar = 10 µm.

We previously reported the introduction of DNA methylation into the ncDNA of pericentromeres in mouse embryos [25]. Generally, mouse oocytes are hypomethylated in centromeres and pericentromeres, as described above. We injected a TALE sequence recognizing the 15 nucleotide (nt) sequence of major satellites (pericentromeres) described by Miyanari et al. [74], fused with SssI. TALE-SssI expression resulted in the successful induction of DNA methylation in target sequences (Figure 3). Using a fluorescent probe for DNA methylation (mCherry–MBD–NLS), upregulated DNA methylation could be observed at the microscopic level, and bisulfite sequencing analysis revealed a statistically significant increase in DNA methylation in major satellites compared with the results from negative controls applying a catalytically dead mutant of SssI. We also applied this technique to embryonic stem (ES) cells lacking all three types of *Dnmt* genes, *Dnmt1*, *Dnmt3a*, and *Dnmt3b*, called *Dnmt* triple KO (TKO) ES cells [75], and successful induction of DNA methylation was clearly observed in nuclei with mCherry–MBD–NLS. Similar results have been obtained using dCas9–SssI with gRNA expression in mouse embryos [25]. As an aid to analyzing the biological functions of germ-cell-specific hypomethylation in centromeres and pericentromeres, we are currently conducting research on early preimplantation embryos and germ cells with this technique. We are conducting studies on the relationship between the developmental capacity of early embryos and centromeric/pericentromeric DNA hypomethylation, and whether embryos with artificially induced DNA methylation in centromeres or pericentromeres develop in vitro and in vivo.

## 7. Conclusions and Perspectives

Regarding the introduction of DNA methylation in mammalian oocytes, it is necessary to consider the reprogramming of DNA methylation that occurs during preimplantation development. Induced DNA methylation may be erased before implantation by active or passive DNA demethylation mechanisms. It is possible that chromosomes have regions suitable for maintaining DNA methylation (e.g., imprinted regions) or easily reprogrammable regions. It is also important to consider how epigenome-editing enzymes are expressed in oocytes. Using the insertion of plasmid DNA sequences into chromosomes, we can expect stable expression, while RNA-mediated expression has transient effects. On the other hand, plasmid injection is risky, as it may lead to the insertion of plasmid DNA into the genome, while RNA-mediated expression has few such risks. It is also necessary to optimize the expression amount of the epigenome-editing enzyme and to design the enzyme and sgRNA (in the case of the CRISPR system) for increased binding specificity to reduce off-target effects. If these problems can be resolved, epigenome editing will create new research fields and medical applications in the future.

There is increasing interest in the developmental origins of health and disease (DOHaD). DOHaD involves abnormal fetal growth and developmental disorders, which are not affected by genetic abnormalities but by epigenetic changes influenced by maternal and paternal nutritional status or stress during pregnancy or gametogenesis [76]. Thus, it has been reported that maternal vitamin C deficiency does not affect embryonic development but is known to cause a reduction in the number of germ cells in the fetus and reduced fertility in adulthood [77]. As for paternal effect, it has also been reported that children whose fathers consumed a diet rich in methyl-donor molecules have altered cognitive and neurological functions [78]. Additionally, mouse pups from fathers eating a diet deficient in folic acid showed a high incidence of congenital anomalies [79]. Because these defects could be caused by epigenetic changes occurring during gametogenesis or preimplantation and postimplantation development, epigenome-editing technology applicable to gametes or embryos is expected to become important not only for basic research but also for studies on DOHaD in human medical research.

## Figures and Tables

**Table 1:** Editing DNA methylation with methyltransferases.

Target	DNA-Binding Module	Effector	References
Maspin	Zinc Finger	DNMT3A CD	[19]
VEGF-A	Zinc Finger	DNMT3A CD-DNMT3L	[20]
HBV x promoter	Zinc Finger	DNMT3A C-term	[21]
Line1	Zinc Finger	MIWI2	[24]
P16 (CDKN2A)	TALE	DNMT3A-DNMT3L	[25]
Major satellite	TALE, dCas9	SssI	[26]
AsclI	TALE-CIB1	DNMT3A CD-CRY2	[27]
BACH-2, IL6ST	dCas9	DNMT3A CD	[18]
Snrpn, CTCF	dCas9	DNMT3A	[28]
Hox genes, Runx1, H19	dCas9	SssI (Q147L)	[29]
SALL2, HBG	dCas9	Split SssI	[30]
HoxA5, KLF4	dCas9-SunTag	scFv-DNMT3A	[31]
IAP (Agouti), H19, IG-DMR, Snrpn DMR	dCas9	DNMT3A	[32]

Maspin: Mammary serine protease inhibitor; VEGF-A: Vascular endothelial growth factor-A; HBV: Hepatitis B virus; Line1: Long interspersed nuclear elements 1; CDKN2A: Cyclin-dependent kinase inhibitor 2A; Ascl1: Achaete-scute homolog 1; BACH2: BTB domain and CNC homolog 2; IL6ST: Interleukin 6 signal transducer; Snrpn: Small nuclear ribonucleoprotein polypeptide N; CTCF: CCCTC-binding factor; Hox gene: Homeobox gene; Runx1: Runt-related transcription factor 1; SALL2: Spalt like transcription factor 2; HBG: Hemoglobin subunit gamma 1; HoxA5: Homeobox A5; KLF4: Kruppel-like factor 4; IAP: Intracisternal A-particle; IG-DMR; Intergenic differentially methylated region; TALE: Transcription activator-like effector; dCas9: nuclease-dead Cas9; CIB1: Calcium and integrin binding 1; SunTag: SUperNovaTag; DNMT3A: DNA methyltransferase 3A; CD: Catalytic domain; DNMT3L. DNA methyltransferase 3 like; C-term: C-terminus; MIWI2: Mouse PIWI 2; CRY2: Cryptochrome circadian regulator 2; Q147L: Glutamine 147 Leucine; scFv: Single-chain variable fragment.

**Table 2:** Editing DNA methylation with ten-eleven translocation (TET) proteins.

Target	DNA-Binding Module	Effector	References
KLF4, RHOX, HBB	TALE	TET1 CD	[17]
ICAM1	Zinc Finger	TET2 CD	[46]
AscI	TALE-CIB1	TET1 CD-CRY2	[27]
Snrpn, BDNF, MyoD	dCas9	TET1 CD	[28]
Gfap, H19 DMR	dCas9-SunTag	scFv-TET1 CD	[41]
BRCA1	dCas9	TET1 CD	[44]
FMR1	dCas9	TET1 CD	[8]
Sox1	dCas9	TET1 CD	[45]
IAP (Agouti)	dCas9	TET1 CD	[32]
hMLH1	dCas9 + gRNA with PUFa-binding site	PUFa-TET1 CD with GADD45A or NEIL2	[42]

KLF4: Kruppel like factor 4; RHOX: Rhox homeobox; HBB: Homoglobin subunit beta; ICAM1: Intercellular adhesion molecule 1; BDNF: Brain derived neurotrophic factor; MyoD: Myogenic differentiation 1; Gfap: Glial fibrillary acidic protein; BRCA1: Breast cancer susceptibility gene 1; FMR1: Fragile X mental retardation 1; Sox1: SRY-box transcription factor 1; hMLH1: Human MutL homolog 1; TET1: Ten-eleven translocation methylcytosine dioxygenase 1; PUF: Pumilio and FBF protein; GADD45: Growth arrest and DNA damage inducible alpha; NEIL2: Nei like DNA glycosylase 2.
