# Erratum: Yamazaki et al. Editing DNA Methylation in Mammalian Embryos. *Int. J. Mol. Sci.* 2020, *21*, 637

**DOI:** 10.3390/ijms22126175

**Published:** 2021-06-08

**Authors:** Taiga Yamazaki, Yu Hatano, Ryoya Taniguchi, Noritada Kobayashi, Kazuo Yamagata

**Affiliations:** 1 Division of Biomedical Research, Kitasato University Medical Center, Kitasato University, 6-100 Arai, Kitamoto, Saitama 364-8501, Japan; kenchu@insti.kitasato-u.ac.jp; 2 Faculty of Biology-Oriented Science and Technology, KINDAI University, 930 Nishimitani, Kinokawa, Wakayama 649-6493, Japan; 1944710002d@waka.kindai.ac.jp (Y.H.); 1933710007k@waka.kindai.ac.jp (R.T.)

The authors wish to make the following corrections to our previously published paper [1]. The correct reference order in Figure 1 should be:

Due to the partially incorrect references shown in the figure and tables, the correct reference order in Table 1 should be:

In Section 2.2, since the reference order has been updated, the correct reference order in this section should be:

When TET1 is used as an effector protein for DNA demethylation, as in DNMT, its catalytic domain is frequently used in epigenome editing [9,26,28,32,41,44,45] (Figure 1 and Table 2). It has also been reported that the catalytic domain of TET2 is fused with zinc finger [46]; 

The application of the SunTag system to tether multiple TET1 proteins to increase the efficiency of DNA demethylation in the target sequence has also been reported [41] (Figure 1 and Table 2);

Morita et al. reported no significant difference in DNA demethylation activity between dCas9–TET1 and dCas9–catalytically dead Tet1 (dTet1) on the *Gfap* and *H19* loci, whereas the dCas9–SunTag system, which enables the tethering of multiple copies of TET1, has strong DNA demethylation activity [41];

On the other hand, other groups have reported that the dCas9–TET1 system is sufficient to decrease DNA methylation in target loci [9,28,32,45,47];

In this system, the effector protein is fused with the Pumilio/FBF RNA-binding domain (PUF domain) and a DNA-binding module comprising a dCas9 and gRNA complex in which multiple PUF binding sites are expressed together with single guide (sg) RNA [48];

Taghbalout et al. reported increased efficiency of DNA demethylation and derepression of genes by multiple tethering of TET1 by a Casilio complex named “Casilio–ME1” compared with the efficiency of DNA demethylation by the dCas9–SunTag system [42].

Due to the partially incorrect references shown in the figure and tables, the correct reference order in Table 2 should be:

**Table 2:** Editing DNA methylation with ten-eleven translocation (TET) proteins.

Target	DNA-Binding Module	Effector	References
KLF4, RHOX, HBB	TALE	TET1 CD	[44]
ICAM1	Zinc Finger	TET2 CD	[46]
AscI	TALE-CIB1	TET1 CD-CRY2	[26]
Snrpn, BDNF, MyoD	dCas9	TET1 CD	[28]
Gfap, H19 DMR	dCas9-SunTag	scFv-TET1 CD	[41]
BRCA1	dCas9	TET1 CD	[45]
FMR1	dCas9	TET1 CD	[9]
Sox1	dCas9	TET1 CD	[47]
IAP (Agouti)	dCas9	TET1 CD	[32]
hMLH1	dCas9 + gRNA withPUFa-binding site	PUFa-TET1 CD with GADD45A or NEIL2	[42]

In Section 4, since the reference order has been updated, the correct reference order in this section should be: 

The application of DNA methylation editing in vivo has also been reported for human clinical therapy in fetal and adult brains [9,41].

In the reference section, the correct reference should be:

41. Morita, S.; Noguchi, H.; Horii, T.; Nakabayashi, K.; Kimura, M.; Okamura, K.; Sakai, A.; Nakashima, H.; Hata, K.; Nakashima, K.; et al. Targeted DNA demethylation in vivo using dCas9-peptide repeat and scFv-TET1 catalytic domain fusions. *Nat. Biotechnol.* **2016**, *34*, 1060–1065.

42. Taghbalout, A.; Du, M.; Jillette, N.; Rosikiewicz, W.; Rath, A.; Heinen, C.D.; Li, S.; Cheng, A.W. Enhanced CRISPR-based DNA demethylation by Casilio-ME-mediated RNA-guided coupling of methylcytosine oxidation and DNA repair pathways. *Nat. Commun.* **2019**, *10*, 4296.

46. Chen, H.; Kazemier, H.G.; de Groote, M.L.; Ruiters, M.H.; Xu, G.L.; Rots, M.G. Induced DNA demethylation by targeting Ten-Eleven Translocation 2 to the human ICAM-1 promoter. *Nucleic Acids Res.* **2014**, *42*, 1563–1574.

47. Baumann, V.; Wiesbeck, M.; Breunig, C.T.; Braun, J.M.; Koferle, A.; Ninkovic, J.; Gotz, M.; Stricker, S.H. Targeted removal of epigenetic barriers during transcriptional reprogramming. *Nat. Commun.* **2019**, *10*, 2119.

48. Cheng, A.W.; Jillette, N.; Lee, P.; Plaskon, D.; Fujiwara, Y.; Wang, W.; Taghbalout, A.; Wang, H. Casilio: A versatile CRISPR-Cas9-Pumilio hybrid for gene regulation and genomic labeling. *Cell Res.* **2016**, *26*, 254–257.

The authors would like to apologize for any convenience caused to the readers by these changes.

## Figures and Tables

**Figure 1:**
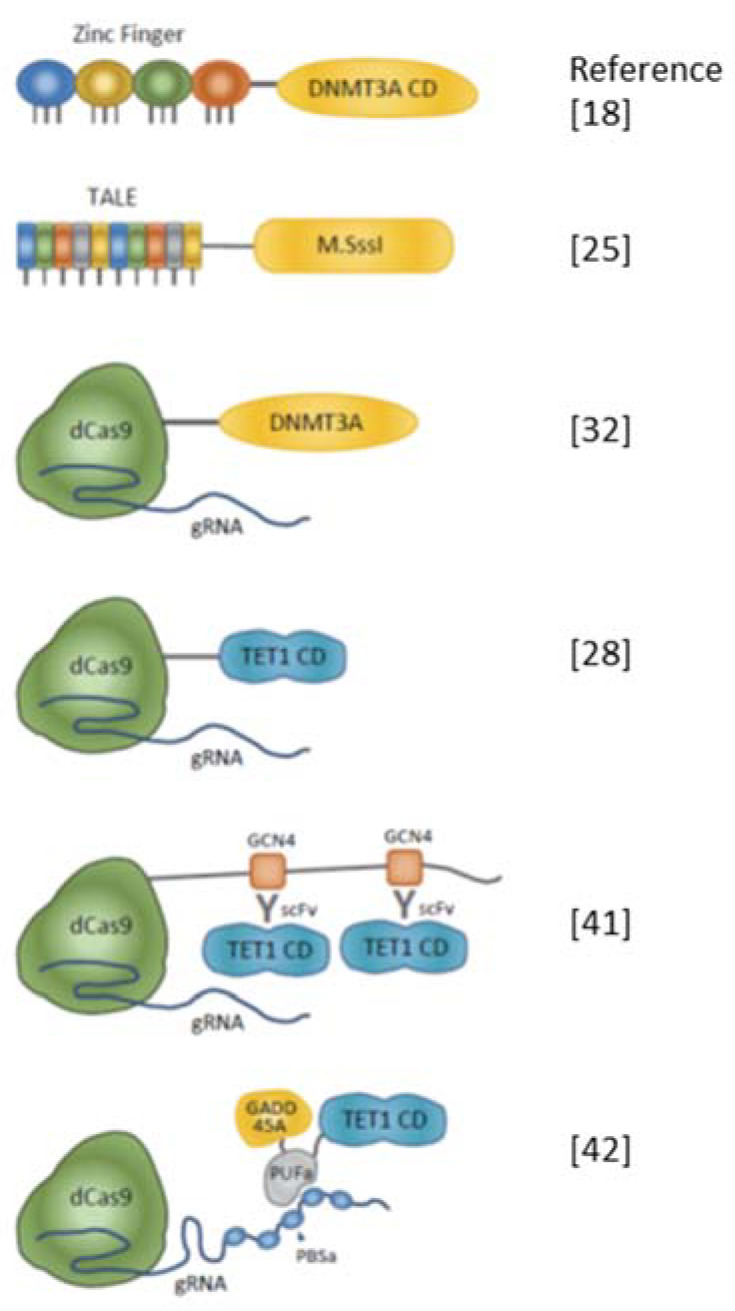
Schematic diagrams of artificial enzymes for editing DNA methylation. Representative combinations of DNA-binding modules and effectors are shown. Zinc finger, transcription activator-like effector (TALE), and dCas9 with guide RNA (gRNA) complex are used for DNA-binding modules. DNMT3A or M.SssI are effectors of inducing DNA methylation. To remove DNA methylation, the catalytic domain (CD) of TET1 is fused with DNA binding module. SunTag technology enables multiple copies of TET1 CD to be introduced to the target region [41]. There has been a report describing the tethering of both TET1 CD and base excision repair (BER)-related proteins such as GADD45A to improve the efficiency of DNA demethylation [42].

**Table 1:** Editing DNA methylation with methyltransferases.

Target	DNA-Binding Module	Effector	References
Maspin	Zinc Finger	DNMT3A CD	[18]
VEGF-A	Zinc Finger	DNMT3A CD-DNMT3L	[19]
HBV x promoter	Zinc Finger	DNMT3A C-term	[20]
Line1	Zinc Finger	MIWI2	[23]
P16 (CDKN2A)	TALE	DNMT3A-DNMT3L	[24]
Major satellite	TALE, dCas9	SssI	[25]
AsclI	TALE-CIB1	DNMT3A CD-CRY2	[26]
BACH-2, IL6ST	dCas9	DNMT3A CD	[27]
Snrpn, CTCF	dCas9	DNMT3A	[28]
Hox genes, Runx1, H19	dCas9	SssI (Q147L)	[29]
SALL2, HBG	dCas9	Split SssI	[30]
HoxA5, KLF4	dCas9-SunTag	scFv-DNMT3A	[31]
IAP (Agouti), H19,IG-DMR, Snrpn DMR	dCas9	DNMT3A	[32]
